# MR1 recycling and blockade of endosomal trafficking reveal distinguishable antigen presentation pathways between *Mycobacterium tuberculosis* infection and exogenously delivered antigens

**DOI:** 10.1038/s41598-019-41402-y

**Published:** 2019-03-18

**Authors:** Elham Karamooz, Melanie J. Harriff, Gitanjali A. Narayanan, Aneta Worley, David M. Lewinsohn

**Affiliations:** 1 VA Portland Health Care System, Research and Development, 3710 SW US Veterans Hospital Road, Portland, 97239 Oregon USA; 2 Pulmonary & Critical Care Medicine, Oregon Health & Science University, 3181 SW Sam Jackson Park Road, Portland, Oregon 97239 USA

## Abstract

The MHC-Ib molecule MR1 presents microbial metabolites to MR1-restricted T cells (MR1Ts). Given the ubiquitous expression of MR1 and the high prevalence of human MR1Ts, it is important to understand the mechanisms of MR1-dependent antigen presentation. Here, we show that MR1-dependent antigen presentation can be distinguished between intracellular *Mycobacterium tuberculosis* (Mtb) infection and exogenously added antigens. Although both Mtb infection and exogenously added antigens are presented by preformed MR1, only exogenously added antigens are capable of reusing MR1 that had been bound to the folic acid metabolite 6-formylpterin (6-FP). In addition, we identify an endosomal trafficking protein, Syntaxin 4, which is specifically involved in the presentation of exogenously delivered antigens but not Mtb-dependent antigen presentation. These data reveal there are multiple ways that MR1 can sample antigens and that MR1-mediated sampling of intracellular Mtb infection is distinguishable from the sampling of exogenously added antigens.

## Introduction

While the regulatory mechanisms governing antigen presentation have been rigorously defined for MHC-I and MHC-II^1^, the molecular mechanisms of antigen presentation by the non-classical class I molecule MR1 are still being elucidated. MR1 is unique because it is monomorphic, it is mostly intracellular with little MR1 expressed on the cell surface, and it presents antigens derived from the microbial metabolome^2^. These features allow MR1 to sample the intracellular environment and present bacterial metabolites to MR1Ts, of which Mucosal-Associated Invariant T (MAIT) cells are a subset. MR1Ts are a highly prevalent CD8 subset that have immediate effector function and are capable of producing pro-inflammatory cytokines against a wide variety of microbes^3^. The ubiquitous expression of MR1 and high prevalence of MR1Ts suggests that the MR1-MR1T cell axis is a critical part of the human immune system. This was illustrated by two recent reports. Seshadri *et al*. showed that an MR1 single nucleotide polymorphism was associated with Mtb meningitis^4^. Second, Pincikova *et al*. described severe, recurrent pulmonary infections in a patient with cystic fibrosis who lacked MAIT cells^5^.

Although the first MR1 antigens identified were derived from riboflavin biosynthesis^6,7^, subsequent studies have defined a broader array of antigens. This includes other microbial metabolites such as photolumazines, non-microbial antigens, synthetic compounds, and an unknown antigen from *Streptococcus pyogenes*, a microbe that does not synthesize riboflavin^8–11^. In addition, there is TCR diversity that is associated with the detection of specific microbes^12^. MR1 antagonists have also been identified, such as 6-FP, which is derived from the photodegradation of folic acid^13^. Despite being an MR1 antagonist, 6-FP induces MR1 translocation to the plasma membrane^14–16^. The crystal structure of MR1 revealed that 6-FP forms a covalent bond with lysine 43 of MR1^3^. The elimination of this positive charge via this bond is thought to be key for MR1 egress from the endoplasmic reticulum (ER) and translocation to the plasma membrane^16^. However, the formation of a covalent bond between lysine 43 and an MR1 antigen is not an absolute requirement for translocation to the plasma membrane because some of the riboflavin derived antigens do not form this covalent bond^17^.

In the study of MR1-dependent antigen presentation, Mtb is an excellent model because antigen presentation in airway epithelial cells is dependent on intracellular infection. Mtb supernatant does not activate MR1Ts. Earlier work demonstrated that intracellular infection was necessary via separate approaches: transwell assays and magnetic bead labelling of Mtb coupled with magnetic sorting of Mtb infected cells^18^. Explanations for this finding could include the possibility that Mtb produces a lower quantity of MR1 antigens, and therefore optimal loading occurs intracellularly where more MR1 molecules are located, or it is possible that Mtb does not secrete MR1 antigens or that they are secreted in the phagosome. We previously showed that in airway epithelial cells, MR1 antigens derived from intracellular Mtb infection could be sampled differently than exogenously delivered ligands^15^. This was based on two findings. First, while knockdown of the endosomal trafficking proteins Syntaxin 18 and VAMP4 both affected MR1-dependent antigen presentation of Mtb to MR1Ts, only Syntaxin 18 knockdown affected 6-FP mediated translocation of MR1 to the cell surface. Second, the addition of 6-FP shortly before the addition of exogenous MR1 antigens resulted in a complete loss of MR1-dependent antigen presentation, whereas Mtb-dependent antigen presentation was intact in the presence of 6-FP^15^. These data suggested distinct MR1 trafficking pathways between exogenously added antigens versus antigen derived from an intracellular infection.

Since MR1 is primarily intracellular, understanding how MR1 detects intracellular microbes is critical to our understanding of MR1 and host defense. We hypothesize that MR1-dependent antigen presentation follows distinct trafficking pathways for intracellular Mtb infection versus exogenously added antigens. In this report, we examine how 6-FP mediated egress of MR1 from the ER impacted antigen presentation of Mtb compared to exogenously added MR1 antigens. We show that overnight incubation with 6-FP boosts the response to exogenously added antigens but has no boosting effect on Mtb. However, both intracellular Mtb infection and exogenously added antigens are capable of being presented by preformed MR1 indicating that new MR1 protein synthesis is not required. The boosting effect also does not depend on new MR1 synthesis but is due in part to 6-FP mediated rescue of MR1 from degradation. Finally, to clearly establish that there are distinct trafficking pathways of MR1, we knocked down endosomal trafficking proteins and found that Syntaxin 4 knockdown affected the presentation of exogenously delivered antigens but has no effect on Mtb-dependent antigen presentation. Collectively, these results demonstrate three important findings: (1) preformed MR1 can present antigens from both Mtb infection and exogenously delivered mycobacterial antigens, (2) 6-FP bound MR1 can be efficiently recycled to present exogenously delivered mycobacterial antigens and (3) the mechanisms of MR1-dependent antigen presentation can differ between exogenously added antigens versus intracellular Mtb infection.

## Results

### 6-FP pretreatment boosts the MR1 response to exogenously added antigens

We previously demonstrated that a short pretreatment of BEAS-2B with 6-FP resulted in substantial inhibition of MR1-dependent antigen presentation of exogenously added antigens while Mtb antigen presentation remained intact. In order to determine whether 6-FP bound MR1 could be competent for antigen presentation, we performed overnight incubation of BEAS-2B with 6-FP. The ability of these cells to present antigen that was either provided exogenously or derived from Mtb infection was tested using IFN-γ release by MR1Ts^19^. We generated dose-response curves by performing serial dilution of antigen presenting cells (APCs) for Mtb infected cells, or serial dilution of exogenously added antigens found in the supernatant from *Mycobacterium smegmatis* (Msmeg). Filtered Msmeg supernatant has a high quantity of MR1 antigens, demonstrated by its performance in IFN-γ ELISPOT assays with MR1Ts, and it retains excellent antigenic activity when stored at −80 C.

**Figure 1:**
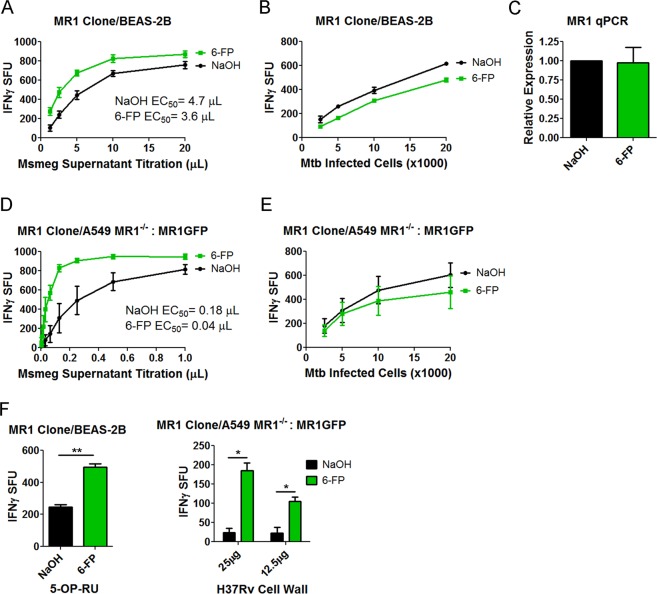
6-FP pretreatment boosts the MR1-dependent response to exogenously added antigens. (**A**,**B**) IFN-γ ELISPOT assay results of BEAS-2B pretreated with 6-FP versus control using (**A**) Msmeg supernatant as the antigen or (**B**) Mtb infection as the antigen. The data are pooled from 3 independent experiments. IFN-γ spot forming units (SFU) are plotted. For (**A**), the difference in the Log EC_50_ was statistically significant between the curves (P = 0.0198). For (**B**), the difference in the Log EC_50_ was not statistically significant between the two curves (P = 0.9629). (**C**) qPCR of MR1 following pretreatment of BEAS-2B with 6-FP or control. The data are pooled from 2 independent experiments. P = 0.1266 (unpaired two-tailed *t*-test). (**D**,**E**) A549 MR1^−/−^: MR1GFP were used as in (**A**,**B**). The data are pooled from 3 independent experiments. For (**D**), the difference in the Log EC_50_ was statistically significant between the two curves (P < 0.0001). For (**E**), the difference in the Log EC_50_ was not statistically significant between the two curves (P = 0.6839). (**F**) IFN-γ ELISPOT assay results of 6-FP pretreatment versus control using 0.1 nM 5-OP-RU (Left) or H37Rv cell wall (Right) as the antigen. Left) P = 0.0087 (unpaired two-tailed *t*-test). Data are representative of 2 independent experiments. Right) P = 0.0191 for the 25 µg condition and P = 0.0461 for the 12.5 µg condition (unpaired two-tailed *t*-test). Data are pooled from 2 independent experiments.

As shown in Fig. 1, we observed that overnight pretreatment with 6-FP significantly boosted MR1-dependent presentation of exogenously added antigens (Fig. 1A). 6-FP pretreatment resulted in a 23% increase in functional avidity as measured by EC_50_^20^. This boosting was not observed with Mtb infection (Fig. 1B). In the absence of antigen, 6-FP pretreatment alone results in no significant IFN-γ release (Supplementary Fig. S1). To determine if 6-FP was altering MR1 transcription, we performed qPCR and found no significant change between the control and 6-FP treated groups (Fig. 1C). To test whether the boosting effect was present in an MR1 overproducing cell, we used a previously described A549 MR1^−/−^ cell line^21^ transduced with a lentivirus expressing MR1GFP^13^ as the APC. These A549 MR1^−/−^: MR1GFP cells produce substantially more MR1 compared to wild type BEAS-2B [Supplementary Fig. S2]. We found that that the A549 MR1^−/−^: MR1GFP cell line resulted in a significantly higher functional avidity for the 6-FP condition compared to control (Fig. 1D). However, these cells showed no boosting effect with Mtb infection (Fig. 1E). Because of the diversity of antigens found in Msmeg and the observation that these antigens differ from MR1 antigens found in *Escherichia coli* (*E. coli*)^8^, we sought to generalize our findings to other exogenous antigens. To do this, we tested whether 6-FP would boost the response to the canonical MR1 ligand 5-OP-RU as well as H37Rv cell wall. H37Rv cell wall contains MR1 antigens and dendritic cells can present these antigens to MR1Ts^19^. We found that presentation was boosted for both antigens with 6-FP pretreatment, indicating that the boosting effect of 6-FP was not limited to Msmeg supernatant (Fig. 1F).

### Creation of a tetracycline inducible MR1GFP construct

**Figure 2:**
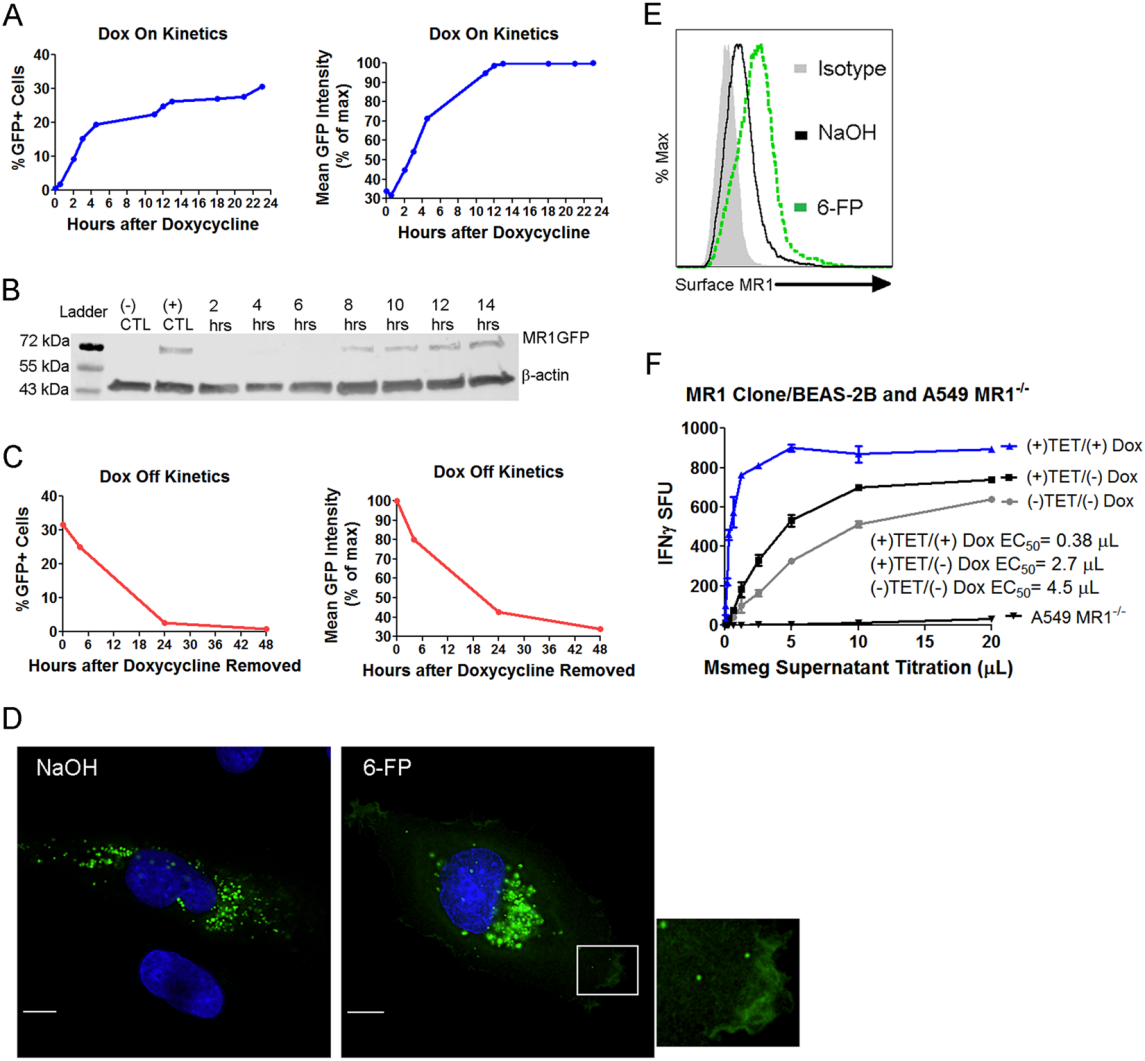
Creation of a tetracycline inducible MR1GFP construct. (**A**) The “on” kinetics of TET-MR1GFP in BEAS-2B measured by flow cytometry. The percentage of GFP positive cells and mean GFP intensity of the GFP positive population normalized to a percentage of the maximum are shown. Data are representative of 2 independent experiments. (**B**) The “on” kinetics of TET-MR1GFP in BEAS-2B measured by western blot. Cropped image is shown (full length blot is presented in Supplementary Fig. S3). Data are representative of 2 independent experiments. (**C**) The “off” kinetics of TET-MR1GFP in BEAS-2B measured by flow cytometry. The percentage of GFP positive cells and mean GFP intensity of the GFP positive population normalized to a percentage of the maximum are shown. Data are representative of 2 independent experiments. (**D**,**E**) BEAS-2B cells transfected with TET-MR1GFP and incubated with doxycycline to induce preformed MR1 were treated with 6-FP or control, after the doxycycline was removed, for 16 hours. Cells were then analyzed by fluorescence microscopy (**D**) or flow cytometry (**E**). (**D**) Green: MR1GFP; blue: nucleus. Scale bars = 10 µm. Images are representative of 3 independent experiments. (**E**) Data are representative of 2 independent experiments. (**F**) IFN-γ ELISPOT assay results of BEAS-2B transfected with TET-MR1GFP using Msmeg supernatant as the antigen. Non-transfected cells ((−)TET/(−)Dox) and A549 MR1^−/−^ served as controls. Data are representative of 2 independent experiments. The difference in Log EC_50_ was statistically significant between the BEAS-2B curves (P < 0.0001).

It has been shown that MR1-dependent antigen presentation remains intact despite treatment with cycloheximide, indicating that an intracellular pool is available to sample antigens and that new protein synthesis is not required^16^. Although our qPCR data did not show an increase in MR1 mRNA with 6-FP, this did not preclude the possibility 6-FP was leading to more translation of MR1 mRNA. While cycloheximide can be used to inhibit protein synthesis, we were concerned about potential off-target effects. Therefore, we generated a tetracycline inducible MR1 construct tagged with GFP, designated TET-MR1GFP, to determine if the boosting effect was due to new MR1 synthesis. In order to quantify the “on” kinetics of this construct, we transfected BEAS-2B with TET-MR1GFP plasmid, treated the cells with doxycycline and performed time course experiments tracking the amount of MR1 by flow cytometry. To do this, we used GFP fluorescence as a surrogate for MR1. Using this approach, we found that approximately 30% of the BEAS-2B cells were transfected with the plasmid and expressing MR1 within 23 hours of addition of doxycycline (Fig. 2A). However, mean fluorescence intensity of the GFP positive population plateaued at 12 hours following the addition of doxycycline.

To confirm that GFP expression correlated with the amount of MR1, we performed time course experiments as in Fig. 2A and utilized western blot to confirm that MR1 was not cleaved from GFP. We used an anti-MR1 antibody and found MR1 protein was detectable at 8 hours after the addition of doxycycline and the band observed was consistent with the predicted molecular weight of MR1GFP at 69 kDa, indicating that the GFP tag had not been cleaved from MR1 (Fig. 2B). To determine how quickly inducible MR1 is degraded, we removed doxycycline at specific time points and analyzed the cells by flow cytometry. Here, we found that little MR1 was present 24 hours after doxycycline is removed (Fig. 2C).

Next, we confirmed that inducible MR1 would have a similar phenotype to constitutively expressed MR1 in the presence and absence of 6-FP. 6-FP mediated stabilization of MR1 can be visualized by microscopy and measured by flow cytometry. We performed fluorescence microscopy of BEAS-2B with inducible MR1 and found that in the absence of 6-FP, there is substantial pool of vesicular MR1 (Fig. 2D). However, with 6-FP treatment, MR1 can be visualized in vesicles and on the cell surface. Flow cytometry confirmed the 6-FP mediated cell surface stabilization of inducible MR1 (Fig. 2E). Finally, to determine whether inducible MR1 could present antigens to MR1Ts, we compared wild type BEAS-2B to BEAS-2B transfected with TET-MR1GFP with or without the addition of doxycycline using an IFN-γ ELISPOT assay as a readout. We observed a substantial increase in MR1-dependent antigen presentation when doxycycline was added to transfected BEAS-2B compared to control conditions (Fig. 2F). Taken together, these data show that inducible MR1 is degraded in the absence of ligand, is located in a vesicular compartment, is able to bind 6-FP and be stabilized on the cell surface, and is competent for antigen presentation to MR1Ts.

### Preformed MR1 bound to 6-FP can present exogenously added antigens

**Figure 3:**
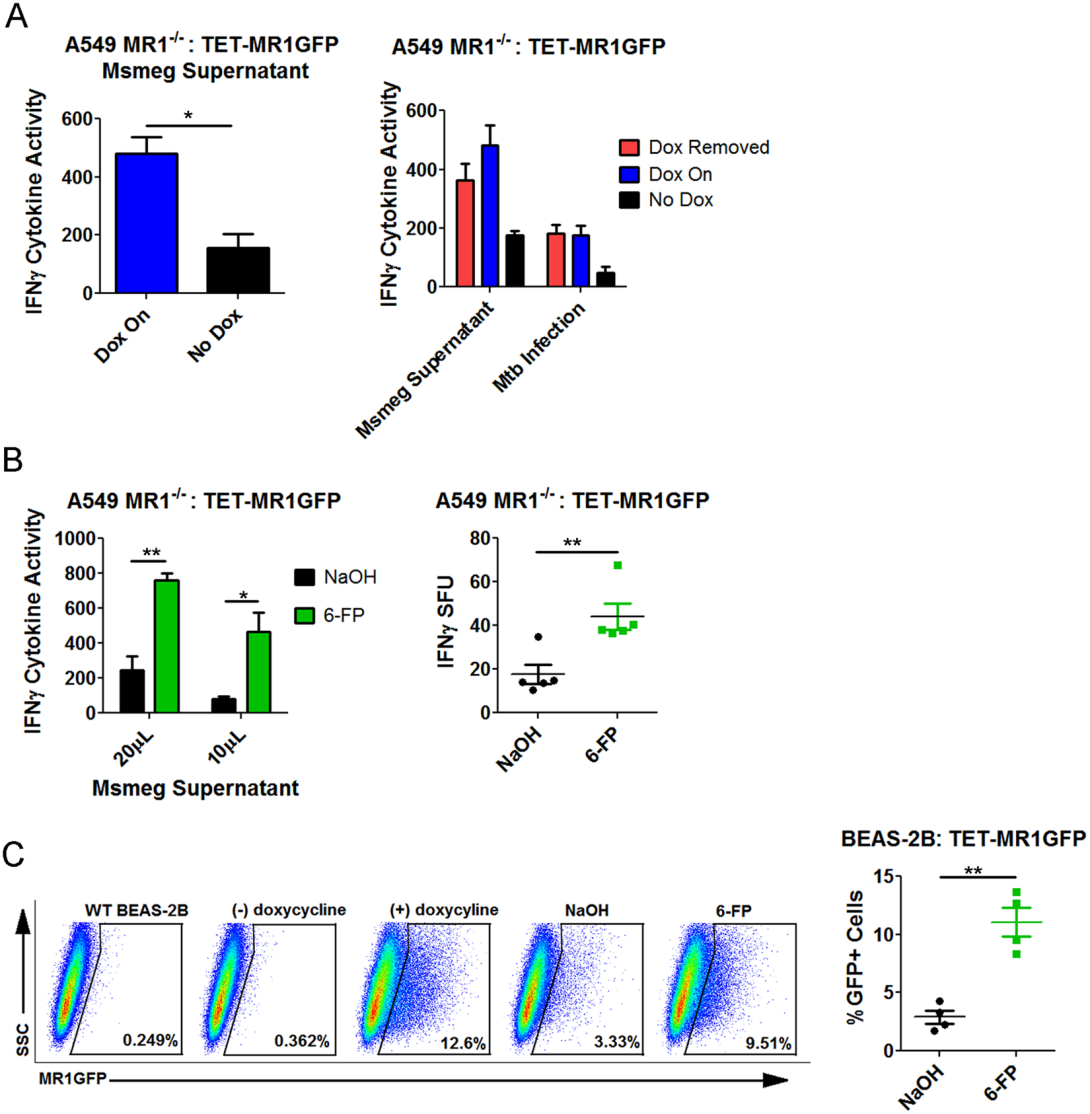
Preformed MR1 bound to 6-FP can present exogenously added antigens. (**A**) Left: A549 MR1^−/−^: TET-MR1GFP were used as APCs in an IFN-γ ELISPOT assay with Msmeg supernatant as the antigen. The data are pooled from 2 independent experiments. An unpaired two-tailed *t*-test was used to calculate statistical significance (P = 0.0493). Right: A549 MR1^−/−^: TET-MR1GFP were used as APCs in an IFN-γ ELISPOT assay with Mtb infection or with Msmeg supernatant comparing preformed MR1 to ongoing MR1 synthesis. Mean and SEM from technical replicates are plotted. Data are representative of 2 independent experiments. (**B**) A549 MR1^−/−^: TET-MR1GFP with preformed MR1 were treated with 6-FP or control and used as APCs in an IFN-γ ELISPOT assay with Msmeg supernatant. Left) plot of IFN-γ cytokine activity. Data are pooled from 3 independent experiments. P = 0.0048 for 20 µL condition and P = 0.0256 for 10 µL condition (unpaired two-tailed *t*-test). Right) pooled data from 5 experiments using 10 µL Msmeg supernatant with IFN-γ SFU plotted. An unpaired two-tailed *t*-test was used to calculate statistical significance (P = 0.0072). (**C**) Flow cytometry of BEAS-2B:TET-MR1GFP with preformed MR1 treated overnight with 6-FP versus control. Left) flow cytometry of a representative experiment. Wild type (WT) BEAS-2B serves as the unstained control. Right) data from 4 independent experiments showing the percent of GFP positive cells. P = 0.0011 (unpaired two-tailed *t*-test).

Our hypothesis for the boosting effect observed with 6-FP was that post-Golgi, recycled MR1 was capable of antigen presentation. Previous work by McWilliam *et al*. showed that MR1 bound to 6-FP could not be loaded with MR1 antigens at the plasma membrane^16^, suggesting any reuse of MR1 involves a recycling mechanism. Although 6-FP forms a covalent bond with MR1, this is a weak bond and we hypothesized that MR1 bound to 6-FP could be reused for presentation of exogenously delivered mycobacterial antigens. To test this hypothesis, we used our inducible MR1 construct to regulate the expression new MR1 in cells in the context of 6-FP. We first assessed whether inducible MR1 could be competent for antigen presentation in A549 MR1^−/−^ by transfecting these cells with TET-MR1GFP and using them as APCs for MR1Ts. We found that when doxycycline is present, the cells were able to present mycobacterial antigen to MR1Ts compared to transfected cells that were never treated with doxycycline (Fig. 3A). In addition, in A549 MR1^−/−^ cells transfected with TET-MR1GFP, preformed MR1 was capable of presenting exogenously delivered antigens as well as antigens derived from Mtb infection (Fig. 3A).

We then examined whether 6-FP pretreatment would boost the MR1-dependent response to exogenously added mycobacterial antigens in TET-MR1GFP transfected A549 MR1^−/−^, where the doxycycline was removed to stop new MR1 synthesis leaving a pool of preformed MR1. Here, there was a significant increase in antigen presentation in the 6-FP treated group (Fig. 3B). Since the doxycycline had been washed away in these cells, MR1 was not actively being synthesized. These data demonstrate that the boosting effect of 6-FP does not depend on new protein synthesis and that a pre-synthesized pool of MR1 that had been bound to 6-FP was capable of binding exogenously added antigens and presenting them to MR1Ts.

Since the boosting effect was unrelated to new MR1 synthesis, we hypothesized that 6-FP pretreatment resulted in a larger pool of MR1 by rescuing MR1 from degradation. This hypothesis was based on the fact the EC_50_ for the MR1 overproducing cell line (Fig. 1D, NaOH condition) was lower than the EC_50_ for the BEAS-2B (Fig. 1A, NaOH condition), which only had native MR1. This difference between the cells implied that more MR1 can lower the EC_50_. To test this hypothesis, we examined whether the addition of 6-FP increased the amount of MR1 available by flow cytometry. Here, doxycycline was removed from BEAS-2B expressing TET-MR1GFP prior to the addition of 6-FP, allowing us to look at the effect 6-FP had on preformed MR1 as opposed to newly synthesized MR1. We found there were significantly more GFP positive cells in the 6-FP condition (Fig. 3C). Taken together, these data add two important findings to our understanding of MR1 trafficking: (1) 6-FP bound MR1 can be efficiently reused for presentation of exogenously added mycobacterial antigens and (2) the mechanism is in part due to 6-FP rescuing MR1 from degradation.

### Knockdown of endosomal trafficking proteins can be used to distinguish Mtb from exogenously antigens

**Figure 4:**
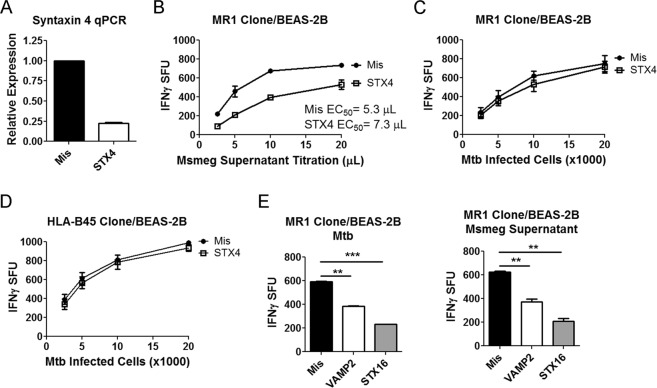
Knockdown of endosomal trafficking proteins can distinguish MR1 trafficking pathways between Mtb and exogenously added antigens. (**A**) qPCR of BEAS-2B after 48 hours of knockdown with missense or Syntaxin 4 (STX4) siRNA. The data are pooled from 3 independent experiments. (**B**) IFN-γ ELISPOT assay results of Syntaxin 4 knockdown in BEAS-2B using Msmeg supernatant as the antigen. Data are pooled from 2 independent experiments. The difference in Log EC_50_ was statistically significant (P = 0.0055). (**C**) IFN-γ ELISPOT assay results of Syntaxin 4 knockdown in BEAS-2B using Mtb infection as the antigen. Data are pooled from 2 independent experiments. The difference in Log EC_50_ was not statistically significant (P = 0.5888). (**D**) IFN-γ ELISPOT assay results of Syntaxin 4 knockdown in BEAS-2B using Mtb infection as the antigen and an HLA-B45-restricted T cell clone. Data are pooled from 2 independent experiments. The difference in Log EC_50_ was not statistically significant (P = 0.7840). (**E**) IFN-γ ELISPOT assay results of VAMP2 and Syntaxin 16 (STX16) knockdown in BEAS-2B. Mean and SEM from technical replicates are plotted. Data are representative of 2 independent experiments. Left) Mtb infection. An unpaired two-tailed *t*-test was used to calculate statistical significance (Mis to VAMP2 P = 0.0011; Mis to Syntaxin 16 P = 0.0003). Right) Msmeg supernatant. An unpaired two-tailed *t*-test was used to calculate statistical significance (Mis to VAMP2 P = 0.0096; Mis to Syntaxin 16 P = 0.0033).

The ability of 6-FP to boost T cell responses against exogenously added antigens but not Mtb added support to our hypothesis that MR1 trafficking pathways can be distinct. We previously demonstrated that knockdown of the endosomal trafficking proteins VAMP4 and Syntaxin 18 affected Mtb-dependent antigen presentation but only Syntaxin 18 knockdown perturbed the 6-FP mediated surface stabilization of MR1. Those data suggested differences in MR1 trafficking between intracellular infection with Mtb and the exogenously added 6-FP ligand. To further test our hypothesis, we performed siRNA knockdown of endosomal trafficking proteins involved in translocation of endosomes to the plasma membrane (VAMP2) and endosomal recycling (Syntaxin 16 and Syntaxin 4) and compared the MR1T responses to Mtb infection versus exogenously added antigens. Syntaxin 4 is implicated in tethering vesicles to the plasma membrane and mediating fusion of recycling endosomes to the plasma membrane^22,23^. We found that knockdown of Syntaxin 4 with siRNA resulted in an 80% decrease in Syntaxin 4 transcripts at 48 hours compared to missense control (Fig. 4A). BEAS-2B that had undergone knockdown of Syntaxin 4 resulted in a decreased response to exogenously added antigens (Fig. 4B). However, knockdown of Syntaxin 4 had no effect on Mtb-dependent antigen presentation (Fig. 4C). Since the Mtb response with Syntaxin 4 knockdown was the same as the missense control, it was unlikely that Syntaxin 4 knockdown perturbed Mtb uptake by BEAS-2B or Mtb viability. However, as a further control, we tested the effect of Syntaxin 4 knockdown on HLA-B45-dependent antigen presentation using an HLA-B45-restricted T cell clone. This T cell clone recognizes a peptide from CFP10_2–9_ and serves as a control for both Mtb uptake and HLA-Ia processing and presentation^24^. We found no change in antigen presentation for Syntaxin 4 knockdown compared to missense control using this HLA-B45-restricted T cell clone (Fig. 4D). On the other hand, knockdown of VAMP2 or Syntaxin 16 affected presentation of Mtb and of exogenously delivered antigens, suggesting that these endosomal trafficking proteins play a shared role in MR1 trafficking (Fig. 4E).

## Discussion

MR1 is a unique non-classical class I molecule in its ability to serve as a sensor of microbial metabolism, potentially allowing for the detection of a wide variety of pathogens. One model of MR1 antigen presentation emphasizes loading in the ER, at which point MR1 associates with β_2_-microglobulin and traffics to the cell surface^2^. Our experiments show that preformed MR1 can present antigens derived from intracellular Mtb infection and exogenously delivered mycobacterial antigens. Moreover, overnight pretreatment with the MR1 antagonist 6-FP boosted the response to exogenously added mycobacterial antigens and the mechanism was due in part to 6-FP rescuing MR1 from degradation. Whether additional mechanisms are at play is unknown. We previously reported that a 2 hr incubation with 6-FP blocked presentation of exogenous antigens. An explanation for this difference is that after prolonged incubation with 6-FP, MR1 undergoes recycling resulting in 6-FP degradation or release. Within this compartment, MR1 is then poised to be reloaded with exogenous antigens. Since 6-FP results in MR1 egress from the ER, these data demonstrate that exogenously delivered mycobacterial antigens can be loaded in a post-Golgi compartment. We postulate that this post-Golgi pathway of MR1-dependent antigen presentation serves as a means of surveilling and sampling endosomal compartments. 6-FP bound MR1 did not boost the response to Mtb but MR1-dependent presentation of intracellular Mtb was dependent on endosomal trafficking as demonstrated by knockdown of VAMP2 and Syntaxin 16. One possible explanation for this difference is that antigens derived from Mtb infection are loaded in a different compartment than exogenously delivered mycobacterial antigens. Several important questions remain including whether MR1 utilizes any chaperone proteins to facilitate antigen loading in the ER or in endosomal compartments and whether MR1 undergoes exchange of ligands or if 6-FP is independently degraded, leaving MR1 available for re-loading.

Another key finding was that blockade of endosomal trafficking proteins revealed distinct pathways for MR1-dependent antigen presentation for intracellular Mtb infection versus exogenously added mycobacterial antigens. We found that Syntaxin 4 knockdown had no effect on Mtb-dependent antigen presentation but significantly impaired the presentation of exogenously delivered antigens. Other data also support distinct trafficking pathways for MR1 between endocytosed microbes and exogenously delivered antigens. Using THP1 cells, Ussher *et al*. showed that blocking endosomal acidification with bafilomycin A1 perturbed the MR1-dependent presentation of fixed intact *E. coli*, but had no effect on presentation of exogenously delivered *E*. coli supernatant^25^. Endosomal trafficking proteins have been used to define distinct pools and distinct pathways of molecules in other biologic systems. For example, in hippocampal cells, the endosomal trafficking protein VAMP4 was found to direct asynchronous calcium release while VAMP2 was important for synchronous calcium release^26^. In adipocytes, VAMP2 was associated with an intracellular pool of GLUT4 that was responsive to insulin while VAMP7 was associated with a pool of GLUT4 that was responsive to osmotic shock^27^.

In a more general context, it is clear that the immune system samples the intracellular environment via a variety of mechanisms. For instance, the Mtb phagosome has been shown to have MHC-Ia and MHC-Ib molecules and the phagosome itself was capable of antigen presentation^28^, demonstrating that additional trafficking of MHC-I molecules, beyond the paradigm of ER to the plasma membrane, is needed to perform intracellular surveillance. Specific endosomal trafficking proteins have been implicated in these processes. For MHC-I, a pool of molecules located in the endosomal recycling compartment was found to be important for cross presentation in professional APCs^28^. This endosomal pool of MHC-I was defined by Rab11a, VAMP3 and VAMP8^29,30^. Even within cross presentation, distinct pathways have been identified, with one pathway requiring TAP and a separate pathway that is TAP independent^31^. In this same study, the data showed that access to these pathways can depend on the physical form of the antigen^31^. Conceptually, distinct antigen presentation pathways such as these may be a way for the immune system to enhance detection of microbes by sampling multiple compartments simultaneously. Along these lines, an early study of MR1 found that MR1 was in a late endosomal compartment in B cells and that MR1 antigen presentation was more closely aligned with MHC-II than MHC-I^32^.

**Figure 5:**
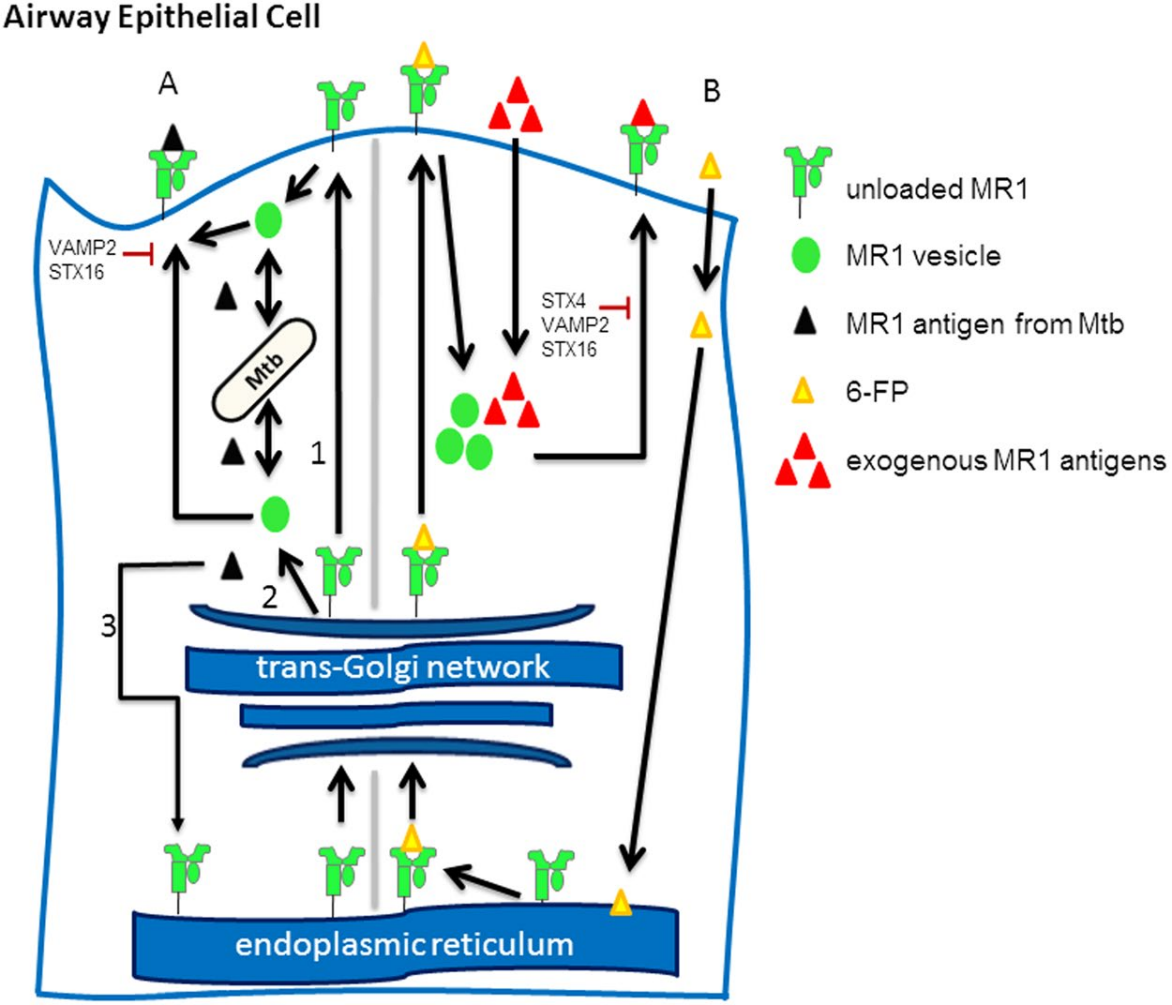
Model for MR1 trafficking. (**A**) MR1 is synthesized in the ER. Some MR1 is able to leave the ER, perhaps with an unidentified endogenous ligand. MR1 captures Mtb antigens via 3 potential pathways: First, MR1 is recycled from the plasma membrane into an endosome that samples the Mtb phagosome. Second, MR1 interacts with Mtb after MR1 exits the trans-Golgi network. Third, MR1 in the ER captures Mtb antigens. Knockdown of the endosomal trafficking proteins VAMP2 and Syntaxin 16 affect Mtb-dependent antigen presentation. (**B**) 6-FP binds MR1 in the ER. 6-FP bound MR1 recycles into a compartment that is suitable for loading of exogenously delivered antigens. After binding exogenous antigens, MR1 traffics back to the cell surface. Syntaxin 4 knockdown blocks the ability of MR1 bound to exogenous antigen to activate T cells. VAMP2 and STX16 also affect presentation of exogenously delivered antigens.

Although we do not know whether there are distinct pools of MR1 for presentation of an intracellular microbe versus exogenously added antigens, we propose a model in which MR1 trafficking between the two can be distinguished (Fig. 5). In this model, 6-FP allows MR1 egress from the ER and stabilization on the cell surface. However, 6-FP stabilized MR1 is then recycled into a compartment that is optimal for loading of exogenously added antigens. Whether 6-FP is actively exchanged with exogenously added antigens, or whether it dissociates from MR1 during internalization is not known. However, it is clear that knockdown of Syntaxin 4 specifically interferes with the ability of MR1 to present exogenously added antigens. In contrast, Mtb-dependent antigen presentation is unaffected by Syntaxin 4 knockdown, but knockdown of VAMP2 and Syntaxin 16 affect both pathways, reflecting perturbation of shared trafficking pathways. Future work will need to focus on the precise mechanisms behind MR1 loading, including any chaperone proteins involved and whether MR1 is stabilized by an endogenous ligand. These important questions must be answered in order to more precisely define the mechanisms of MR1-dependent antigen presentation.

## Materials and Methods

### Bacteria and cells

Mtb (H37Rv, ATCC) was grown in Middlebrook 7H9 broth supplemented with Middlebrook ADC (ThermoFisher Scientific), 0.05% Tween-80, and 0.5% glycerol. The bacteria were passaged 10 times through a tuberculin syringe before infection. MOI of 8 was used for all experiments. Msmeg was cultured with shaking for 24 hrs then pelleted. The supernatant was passed through a 0.22 µm filter. The supernatant was aliquoted and stored at −80 C. BEAS-2B were obtained from ATCC. A549 MR1^−/−^: MR1GFP cells were made by transducing A549 MR1^−/−^ ^20^ with MR1GFP lentiviral particles produced by the University of Pennsylvania Vector Core^15^. All cell lines were cultured in DMEM (Gibco) supplemented with L-glutamine and 10% heat inactivated FBS. All experiments with Mtb were done in a Biosafety Level 3 laboratory. Waste was decontaminated in 3% Wescodyne and autoclaved. All other experiments were done in a Biosafety Level 2 laboratory.

### Reagents and antibodies

Doxycycline was suspended at 2 mg/mL in sterile water and used at 2 µg/mL. 6-formylpterin (Schircks Laboratories) was suspended at 3 mg/mL in 0.01M NaOH and used at 100 µM. The control was an equal volume of 0.01 M NaOH. The precursor 5-N-RU was synthesized by the OHSU Medicinal Chemistry Core. 5-N-RU was converted to 5-A-RU via previously published methods^7^. 5-OP-RU was synthesized by adding 5 molar equivalents of methylglyoxal to 1 molar equivalent of 5-A-RU in a buffer of 100 mM HEPES and 150 mM NaCl, pH 7.2 (final concentration was based on theoretical yield). H37Rv cell wall (BEI Resources) was diluted in PBS to 5 µg/mL. The following antibodies were used for western blots: ab55164, mouse monoclonal to MR1 (Abcam), ab8227, rabbit polyclonal to β-actin (Abcam), goat anti-mouse 800 nm (LI-COR) and goat anti-rabbit 700 nm (LI-COR). The following antibodies were used for flow cytometry: anti-MR1 26.5 (gift from Ted Hansen, biotinylated by BioLegend), streptavidin-Alexa 647 (ThermoFisher Scientific), and biotinylated mouse anti IgG2A (BioLegend). Human serum was obtained from donors. This study was conducted according to the principles expressed in the Declaration of Helsinki. Study participants, protocols and consent forms were approved by Oregon Health & Science University Institutional Review Board (IRB00000186). Written and informed consent was obtained from all donors.

### siRNA knockdown

BEAS-2B plated in 6-well tissue culture plates (Corning) at 70% confluency were transfected with 50 nM siRNA using HiPerfect (Qiagen). siRNA was obtained from ThermoFisher Scientific: Syntaxin 4 s13595, Syntaxin 16 s16528, VAMP2 s13667, and Missense (silencer select negative control No. 1). Knockdown was done for 48 hours.

### ELISPOT assays

ELISPOT assays were done using 96 well MSHA plates (Merck Millipore) and coated with an antibody against IFN-γ (Mabtech). For assays using Msmeg supernatant, APCs were plated in 6-well tissue culture plates and treated with 6-FP versus control. After 16 hrs, the cells were harvested using RPMI (Gibco) supplemented with L-glutamine, 10% heat inactivated human serum and gentamicin. The cells were used as APCs in an IFN-γ ELISPOT assay (1e4 cells/well, plated in duplicate). Msmeg supernatant was added as a serial dilution with total volume kept constant. After 1hr, the MR1-restricted T cell clone D426-G11^12,18^ was added at 1e4 T cells/well. This T cell clone is TRAV1-2 positive. After overnight incubation at 37C and 5% CO_2_, the plate was developed with ALP antibody (Mabtech). IFN-γ SFU were measured on an AID ELISPOT reader. The mean of technical replicates was used to pool data from different experiments. ELISPOT assays using 5-OP-RU and H37Rv cell wall were done as discussed above with serial dilution of antigen. For ELISPOT assays using Mtb, APCs were plated in 6-well tissue culture plates and treated with 6-FP versus control. After 16 hrs, the cells were infected with Mtb. After 16 hrs, the cells were used in an IFN-γ ELISPOT assay, with serial dilution of the APCs. T cell clone D426-G11 was added at 1e4 cells/well. For knockdown experiments, the cells were infected with Mtb after 48 hrs of knockdown. The cells were then used as APCs in an IFN-γ ELISPOT assay with 1e4 MR1-restricted T cells (D426-G11) or HLA-B45-restricted T cells (D466-A10)^24^.

### RNA isolation, cDNA synthesis and qPCR analysis

Total RNA was isolated using RNeasy Mini Kit (Qiagen). cDNA was synthesized using a High Capacity cDNA Reverse Transcription Kit (ThermoFisher Scientific). qPCR was performed using TaqMan Universal PCR Master Mix (ThermoFisher Scientific) on a Step One Plus Real-Time PCR System (Applied Biosystems). FAM-MGB TaqMan Gene Expression Assays for all targets were obtained from ThermoFisher Scientific. Reactions were run in triplicate and data normalized to GAPDH. Expression levels were determined using the 2^−ΔΔCT^ method.

### Construction of TET-MR1GFP and transfection

A tetracycline inducible promoter was designed with a CMV minimal promoter, a human ubiquitin promoter and a reverse transactivator sequence (ThermoFisher Scientific). The sequence was flanked with AscI and NotI restriction sites and was cloned into the plasmid PCI ASCI, which is our previously described PCI plasmid^15^ with an AscI cut site upstream of the CMV promoter. The new construct is designated PCI ASCI TET. MR1GFP was ligated into this construct using EcoRI and KpnI to create TET-MR1GFP. Confirmatory sequencing was performed. Restriction enzymes and ligation kit were obtained from New England Biolabs. PCR and gel purification kits were obtained from Qiagen. Transfection of TET-MR1GFP was done using an Amaxa Nucleofector, Kit T solution (Lonza). Each transfection reaction was done with 1e6 cells and 6 µg of TET-MR1GFP plasmid.

### TET-MR1GFP flow cytometry and ELISPOT assays

To study the kinetics of TET-MR1GFP when doxycycline is added, BEAS-2B were transfected with TET-MR1GFP and treated at specific time points with doxycycline. The cells were fixed in 1% PFA and analyzed with a BD FACSCanto II flow cytometer and FACS Diva software. All analyses were performed using FlowJo software. To study the kinetics when doxycycline is removed, BEAS-2B transfected with TET-MR1GFP were treated with doxycycline for 24 hrs. The doxycycline was removed at specific time points by aspirating the media and washing three times with media. The cells were fixed and analyzed by flow cytometry as described earlier. To study the effect of 6-FP on MR1 half-life, BEAS-2B transfected with TET-MR1GFP were treated with doxycycline, with the exception of the negative control and non-transfected cells. After 24 hrs, the doxycycline was removed and the cells were treated with 6-FP versus control. The following day, the cells were fixed and analyzed by flow cytometry. The positive control represents transfected cells with continuous doxycycline exposure.

To study antigen presentation of TET-MR1GFP, BEAS-2B transfected with TET-MR1GFP were treated with doxycycline with the exception of the no doxycycline control, (+)TET/(−)Dox. Non-transfected BEAS-2B, designated (−)TET/(−)Dox, and A549 MR1^−/−^served as an additional control. After 24 hrs, the cells were used in an IFN-γ ELISPOT assay. To study the TET-MR1GFP construct in A549 MR1^−/−^, the cells were transfected and treated with doxycycline, with the exception of the no doxycycline control. After 24 hrs, the cells were used in an IFN-γ ELISPOT assay (2e4 APC/well, 20 µL Msmeg supernatant). To study preformed MR1, A549 MR1^−/−^ transfected with TET-MR1GFP were treated with doxycycline for 24 hrs. Doxycycline was removed from one condition. The cells were infected with Mtb or left overnight to be used in an IFN-γ ELISPOT assay with Msmeg supernatant (3e4 APC/well, 20 µL Msmeg supernatant). To test the effect of 6-FP pretreatment, A549 MR1^−/−^ transfected with TET-MR1GFP were treated with doxycycline for 24 hrs. The doxycycline was then removed and the cells were treated with 6-FP versus control. The next day, the cells were used in an IFN-γ ELISPOT assay.

### Western blot

BEAS-2B transfected with TET-MR1GFP were treated with doxycycline at specific time points with the exception of the negative control. The positive control represents 24 hrs of doxycycline exposure. Cells were suspended in sample buffer, boiled at 100 C for 5 min and loaded on a Mini-Protean TGX 4–20% gel (Bio-Rad). The gel was run at 100 V and transferred to a PVDF membrane. The membrane was blocked for 1 hour with Odyssey buffer (LI-COR), and then stained with 0.5 µg/mL of ab55164 and 0.04 µg/mL of ab8227 in Odyssey buffer with 0.1% Tween 20. Secondary staining was done with 1:5000 dilutions of goat anti-mouse 800 nm and goat anti-rabbit 700 nm. The membrane was imaged on an Odyssey CLx imager (LI-COR).

### MR1 surface stabilization

BEAS-2B transfected with TET-MR1GFP were plated into 6-well tissue culture plates. The next day, the cells were treated with 6-FP versus control. After 16 hrs, the cells were harvested on ice and split into two groups for primary staining and isotype control staining. Primary staining was done with an antibody against MR1 at 1:100 for 40 min on ice in the presence of 2% human serum, 2% goat serum, and 0.5% FBS. Biotinylated mouse anti IgG2A served as the isotype control. After washing, streptavidin-Alexa 647 was added for 40 min on ice. Cells were washed, fixed and analyzed by flow cytometry.

### Fluorescence microscopy

BEAS-2B transfected with TET-MR1GFP were plated into 1.5 mm glass bottom chamber slides (Nunc) and incubated at 37C and 5% CO_2_. After 24 hrs of doxycycline treatment, the doxycycline was removed and the cells were treated with 6-FP versus control. After 16 hrs, the cells were stained with NucBlue Live Cell Stain (ThermoFisher Scientific). Images were acquired on a high-resolution wide field CoreDV system (Applied Precision) with a Nikon Coolsnap ES2 HQ. Each image was acquired as Z-stacks in a 1024 × 1024 format with a 60x objective (NA 1.42). Images were processed on Imaris (Bitplane).

### Data analysis

Data were analyzed with Prism 5 GraphPad Software. Statistical significance was determined using unpaired Student’s two-tailed *t*-test. For measurements of functional avidity, the curves were transformed to a semilog scale and normalized. Best-fit EC_50_ was calculated.

## Supplementary information

Supplementary Information

## Data Availability

All data generated and analyzed during this study are included in this published article.
