# Supplementary material for: MR1 recycling and blockade of endosomal trafficking reveal distinguishable antigen presentation pathways between Mycobacterium tuberculosis infection and exogenously delivered antigens
Source: Sci Rep. 2019 Mar 18;9:4797. doi: 10.1038/s41598-019-41402-y (PMC6423294; doi:10.1038/s41598-019-41402-y)
Supplement: Supplementary file 1 — Supplementary Information [file 41598_2019_41402_MOESM1_ESM.docx]

**Title**

MR1 recycling and blockade of endosomal trafficking reveal distinguishable antigen

presentation pathways between *Mycobacterium tuberculosis* infection and exogenously

delivered antigens

**Authors**

Elham Karamooz1,2,*, Melanie J. Harriff1,2, Gitanjali A. Narayanan 2, Aneta Worley2,

David M. Lewinsohn1,2,*

*corresponding authors

**Supplementary Figure S1: 6-FP has no effect on IFN-γ release by the MR1 clone D426-G11**.

BEAS-2B pretreated with 6-FP or control vehicle were used at 10,000 antigen presenting cells per well in the absence of any MR1 antigens. D426-G11 MR1-restricted T cells were added at 10,000 per well. Phytohemagglutin (PHA) is shown as the control for T cell activation. Mean and SEM from technical replicates are plotted.

**Supplementary Figure S2: Relative expression of MR1 in A549 MR1^-/-^ : MR1GFP compared to BEAS-2B.**

qPCR of MR1 was performed as discussed in Figure 1C. The amount of MR1 from BEAS-2B was normalized to 1.

**Supplementary Figure S3: Full-length blot of Figure 3B.**
